# A benchmark dataset for analyzing and visualizing the dynamic epiproteome

**DOI:** 10.1016/j.dib.2019.104000

**Published:** 2019-05-30

**Authors:** Sandeep Kaur, Benedetta Baldi, Jenny Vuong, Seàn I. O'Donoghue

**Affiliations:** a University of New South Wales, Australia; b Garvan Institute of Medical Research, Australia; c Commonwealth Scientific and Industrial Research Organisation, Australia

## Abstract

In this paper, we present a benchmark dataset to evaluate the currently available analysis methods and visualizations for epiproteomic data. The benchmark dataset is a subset of a high-throughput time-series study of phosphoevents occurring upon insulin stimulation. Our dataset is provided in multiple formats for use with four currently available tools. We also provide a file containing the kinase assignments for the sites, as well as a simple kappa model on phosphorylation changes in insulin signalling. A detailed description of the tools, their analysis methods, and the visualizations generated using the input files described here, are discussed in detail in the accompanying review titled “Visualization and analysis of epiproteome dynamics" [1].

**Table:** Specifications Table

Subject area	*Biology*
More specific subject area	*Proteomics*
Type of data	*Text files, Excel file, Python scripts, and Kappa model*
How data was acquired	*103 profiles were selected from a previously published dataset of 37,000 profiles acquired via SILAC mass-spectrometry. The model data and the kinase assignments were derived by surveying the literature.*
Data format	*Comma separated value file; tab separated text files; python script; kappa model; excel file.*
Experimental factors	*Causative kinases for the observed phosphorylations were gathered through literature survey.*
Experimental features	*103 phosphoproteomics profiles were converted into multiple formats for use with multiple tools. The 103 profiles are derived from a much larger dataset by Humphreyet* *al.2013 who measured phosphorylation changes in response to insulin stimulation at 9 time points.*
Data source location	*Garvan Institute of Medical Research, Sydney Australia*
Data accessibility	*Data are provided with this article as supplementary material, and can also be found in a github repository at:**https://github.com/ODonoghueLab/PhosToolInputs*
Related research article	*Kaur S, Baldi B, Vuong J and O'Donoghue SI. Visualization and Analysis of Epiproteome Dynamics. Journal of molecular biology 2019.* doi: https://doi.org/10.1016/j.jmb.2019.01.044[1].

**Table:** 

**Value of the data** • We present a benchmarking dataset to evaluate tools, analysis methods and visualizations for the dynamic epiproteome. The phosphosites in this dataset are well studied - they have been the subject of multiple prior publications. • We provide these data converted into the required input formats for four currently available tools. These files can be used to recreate visualizations discussed in the originating paper. • We provide a small kappa model on the subject of these phosphosites. Models such as these can be used to validate our understanding of the processes in the underlying cellular system.

## 1 Data

In this paper, we provide a benchmark dataset for evaluating the analysis and visualization methods enabled by currently available tools [1]. This dataset contains 103 profiles chosen from Humphrey *et* *al.* (2013) [2]. A summary of the input files and tools is provided in Table 1.

**Table 1:** Summary of the input files provided for each tool.

Input file provided	Format	Tool name	Tool link
DiBS/Dibsvis.csv	CSV file	DiBS	http://www.dibsvis.com/biowheel
DynaPho/DynaPho.txt	Tab separated file	DynaPho	http://140.112.52.89/dynapho/
1. PhosphoPath/PhosphoPath_network.txt	1. Tab separated file	PhosphoPath	http://apps.cytoscape.org/apps/phosphopath
2. PhosphoPath/PhosphoPath_timeseries.txt	2. Tab separated file		
Phoxtrack/Phoxtrack.txt	Tab separated file	Phoxtrack	http://phoxtrack.molgen.mpg.de
Kappa/InsulinSignallingModel.ka	Kappa format	Kappa	https://kappalanguage.org (and follow the link to OnlineUI)

## 2 Experimental design, materials and methods

Humphrey *et* *al.* [2] carried out a comprehensive study measuring phosphorylation changes in response to insulin signalling in 3T3-L1 mouse cells. They published a dataset containing 37,248 distinct phosphorylation events involving 5705 distinct proteins across 9 time points (including basal). The incidence of similar datasets, measuring dynamic epiproteomic changes, is increasing [3], [4], [5].

Thus, from Humphrey *et* *al.* we chose 103 profiles as a benchmarking dataset for evaluating analysis and visualizations possible through available tools for epiproteomics data. Such a selection had many advantages: a dataset of 103 profiles is easily managed compared to the total of 37,248. Furthermore, the phosphosites in this dataset are well studied, as they have been the subject of Figure 5 in Humphrey *et* *al.*, and in subsequent publications [6], where the signalling networks underlying these observed data have been well documented and an assignment of kinases responsible for the phosphorylations presented (in the file Kinase_assignments.xlsx).

In the originating paper [1], we undertook a review of the various tools and methods for visualizing and analyzing dynamic epiproteomic datasets. We utilized the benchmark dataset for this purpose by converting it into the formats required by the tools. Thus, in this paper, we provide six input files that can be used to create visualizations with five tools.

The 103 profiles from Humphrey *et* *al.* are provided in the file Original.txt. This dataset was converted to various input formats for use with the tools: DiBS, DynaPho, PhosphoPath and PHOXTRACK. Additionally, to demonstrate the use of mathematical modelling to evaluate our understanding of these data, we created a sample kappa model on phosphorylation changes in insulin signalling by surveying the literature, and provide it for use with kappa tools. The input files for these five tools are described in the sections below.

### 2.1 DiBS

Input for this tool is provided in the file Dibsvis.csv. It is a simple CSV file containing the columns ‘Gene name’, ‘Phosphorylated amino acid’, ‘IPI position’ (where IPI is the International Protein Identifier) and the abundance ratios at 8 time points.

### 2.2 DynaPho

Input for this tool is provided in the file Dynapho.txt. It is a tab-separated file containing the human UniProt identifier, site (phosphosite position), residue (amino acid), 13-amino acid peptide sequence, and the abundance ratios at 8 time points. All the columns were directly extracted from the original dataset, except for UniProt identifier and site. This is because DynaPho only accepts human data, therefore, the mouse proteins and the phosphosites were converted to their human equivalents by performing a BLAST search [7] of each mouse protein against all human proteins in UniProt.^1^

### 2.3 PhosphoPath

Two input files are required for creating a visualization using this tool. Thus, two files are provided, namely, PhosphoPath_network.txt and PhosphoPath_timeseries.txt.

The file PhosphoPath_network.txt provides a network structure for representing a protein and its phosphosites. Since PhosphoPath is a plugin for Cytoscape, where a network is represented in the form ‘source node’ and ‘target node’, the same structure is used here: the protein node as a source node and the site node as a target node, in columns 1 and 2, respectively. Columns 3 and 4, contain the site node display name and the peptide number, respectively.

The file PhosphoPath_timeseries.txt contains time series values, which are used to build the heatmap in the visualization (see Figure 5 in the originating article). These data are in two columns, where the first column is in the format “UniProt Id-Residue Site-Peptide Id-Time point number”, and the second column contains the quantified abundance ratio value at that particular time point for the phosphosite. We additionally also provide a script (getTpPhosData.py) and an example input file (phosphopath_input.tab) which can be used to generate this file. Details needed to run this script are found in the README.md file.

### 2.4 Phoxtrack

Data for this tool are provided in the file Phoxtrack.txt. It is a tab-separated file containing the 13-amino acid peptide sequence, followed by the mass spectrometry abundance ratios at 8 time points.

### 2.5 Kappa

We provide a model of phosphorylation changes in response to insulin stimulation in the Kappa language format, in the file InsulinSignallingModel.ka. This model was compiled from the literature. It does not aim to provide an accurate representation of the underlying biology, but was built only for demonstration purposes (to demonstrate the use of Kappa tools for epiproteomics data, see section Kappa in the originating article).

The model contains 13 proteins, 2 additional molecules (GTP and GDP), and a total of 33 reactions. The initial concentration of all molecules was set to 10, and the concentration of GTP was set to 1000, GDP was set to 10 (bound to ras protein). We observed the output of Insr Y1175 and Erk1 T203. A list of modelled reactions are shown in Table 2.

**Table 2:** Reactions modelled in the InsulinSignallingModel.ka file.

Reaction name in the model	Reaction details
ins_insr_binding	Binding between insulin and the alpha subunit of the insulin receptor [8]
insr989_autophos, insr1175_autophos, insr1179_autophos, insr1180_autophos	Autophosphorylation of Insr at sites Y989, Y1175, Y1179 and Y1180, respectively [9]
insr_irs1_binding	Binding of Irs1 to Insr [9]
irs1_y460, irs1_y608, irs1_y628, irs1_y891, irs1_y935, irs1_y983, irs1_y1171, irs1_y1218	Phosphorylation of Irs1 at sites Y460 [10], Y608 [10], Y628 [10], Y891 [11], Y935 [10], Y983 [10], Y1171 [11] and Y1218 [11], respectively, by Insr
irs1_grb2	Binding of Irs1(at Y891) and grb2 [11], [12]
shc1_y423	Shc1 Y423 phosphorylation by Insr [13]
shc1_grb2	Phosphorylated Sch 1 at Y423 binding to Grb2 [12]
grb2_sos	Grb2 (bound to shc1) binding to Sos [14]
sos_GDP	When Grb2 bound to Shc1, GDP bound to Ras unbinds [14]
ras_GTP	Ras binding to GTP [14]
ras_raf1	Ras is binding to Raf1 [14]
map2k1_s218, raf1_map2k1	Phosphorylation of Map2k1 at sites S218 and S222, respectively, by raf1 [15]
map2k2_s222, raf1_map2k2	Phosphorylation of Map2k2 at sites S222 and S226, respectively, by raf1 [15]
erk1_t203, erk1_y205, erk2_t183, erk2_y185	Phosphorylation of Erk1 and Erk2 at sites T203 and Y205, and T183 and Y185 respectively, by Map2k1 [2]
erk1_t203_b, erk1_y205_b, erk2_t183_b, erk2_y185_b	Phosphorylation of Erk1 and Erk2 at sites T203 and Y205, and T183 and Y185 respectively by Map2k2 [2]

### 2.6 Benchmark dataset limitations

In this paper, a benchmark dataset is presented, in various formats, consisting of profiles of 103 phosphosites on 58 proteins, and their associated kinases. Although the sites in this dataset are well studied, a number of limitations are present.

The original study by Humphrey *et* *al.* [2], which this benchmark dataset is derived from, was considered a cutting-edge landmark study in 2013. However, mass spectrometry technologies are improving every year, thus, now, five years on, if the study is repeated, due to advances in technologies it is possible that the results may differ and offer higher resolution, sensitivity, and coverage.

Secondly, of the 37,000 sites quantified, the benchmark dataset is a very small subset with only 103 sites. Furthermore, even though these 103 sites have been well studied and depict both phosphorylation and dephosphorylation changes, greater emphasis has been laid on studying the phosphorylation changes, and thus the phosphatases potentially causing the dephosphorylation changes are not included in our benchmark. Instead, dephosphorylation was considered only indirectly, as a result of deactivation of the kinases [6].

Finally, in spite of the focus on phosphorylation events, even for the well-studied 103 sites, not all causative kinases are known. These are presented in the kinase assignments file (Kinase_assignments.xlsx) as “?”. Furthermore, while the kinases included in the benchmark are well known and the assignments are based on good evidence, there is still the possibility that some of these assignments are not entirely accurate since, for example, the evidence may be generated under different experimental conditions or in different cell lines.

## Footnotes

1 UniProt database website: https://www.uniprot.org/, last visited on 2019, March 28.
